# Photoperiodic diapause in a subtropical population of *Aedes albopictus* in Guangzhou, China: optimized field-laboratory-based study and statistical models for comprehensive characterization

**DOI:** 10.1186/s40249-018-0466-8

**Published:** 2018-08-14

**Authors:** Dan Xia, Xiang Guo, Tian Hu, Li Li, Ping-Ying Teng, Qing-Qing Yin, Lei Luo, Tian Xie, Yue-Hong Wei, Qian Yang, Shu-Kai Li, Yu-Ji Wang, Yu Xie, Yi-Ji Li, Chun-Mei Wang, Zhi-Cong Yang, Xiao-Guang Chen, Xiao-Hong Zhou

**Affiliations:** 1 Department of Pathogen Biology, Key Laboratory of Prevention and Control for Emerging Infectious Diseases of Guangdong Higher Institutes, Guangdong Provincial Key Laboratory of Tropical Disease Research, School of Public Health, Southern Medical University, Guangzhou, 510515 Guangdong China; 2 WHO Collaborating Centre for Infectious Disease Epidemiology and Control, School of Public Health, Li Ka Shing Faculty of Medicine, The University of Hong Kong, Hong Kong, Special Administrative Region China; 3 Guangzhou Center for Disease Control and Prevention, Guangzhou, 510515 China

**Keywords:** Subtropical, *Aedes albopictus*, Critical photoperiod, Photoperiodic diapause, Distributed lag non-linear model

## Abstract

**Background:**

*Aedes albopictus* is among the 100 most invasive species worldwide and poses a major risk to public health. Photoperiodic diapause provides a crucial ecological basis for the adaptation of this species to adverse environments. *Ae. albopictus* is the vital vector transmitting dengue virus in Guangzhou, but its diapause activities herein remain obscure.

**Methods:**

In the laboratory, yeast powder and food slurry were compared for a proper diapause determination method, and the critical photoperiod (CPP) was tested at illumination times of 11, 11.5, 12, 12.5, 13, and 13.5 h. A 4-parameter logistic (4PL) regression model was selected to estimate the CPP. In the field, the seasonal dynamics of the *Ae. albopictus* population, egg diapause, and hatching of overwintering eggs were investigated monthly, weekly, and daily, respectively. A distributed lag non-linear model (DLNM) was used to assess the associations of diapause with meteorological factors.

**Results:**

In the laboratory, both the wild population and the Foshan strain of *Ae. albopictus* were induced to diapause at an incidence greater than 80%, and no significant difference (*P* > 0.1) was observed between the two methods for identifying diapause. The CPP of this population was estimated to be 12.312 h of light. In the field, all of the indexes of the wild population were at the lowest levels from December to February, and the Route Index was the first to increase in March. Diapause incidence displayed pronounced seasonal dynamics. It was estimated that the day lengths of 12.111 h at week_2016, 43_ and 12.373 h at week_2017, 41_ contributed to diapause in 50% of the eggs. Day length was estimated to be the main meteorological factor related to diapause.

**Conclusions:**

Photoperiodic diapause of *Ae. albopictus* in Guangzhou of China was confirmed and comprehensively elucidated in both the laboratory and the field. Diapause eggs are the main form for overwintering and begin to hatch in large quantities in March in Guangzhou. Furthermore, this study also established an optimized investigation system and statistical models for the study of *Ae. albopictus* diapause. These findings will contribute to the prevention and control of *Ae. albopictus* and mosquito-borne diseases.

**Electronic supplementary material:**

The online version of this article (10.1186/s40249-018-0466-8) contains supplementary material, which is available to authorized users.

## Multilingual abstract

Please see Additional file 1 for translations of the abstract into the five official working languages of the United Nations.

## Background

*Aedes albopictus* (Skuse) is one of the 100 most invasive species worldwide [1]. Due to increased international trade, population flows, global warming, and accelerating urbanization, the distribution of *Ae. albopictus* has been expanding rapidly. Native to tropical Southeast Asia islands of the western Pacific and the Indian Ocean, *Ae. albopictus* has colonized all continents except mainland Australia and Antarctica within the last 30–40 years and has become widely distributed in temperate, subtropical, and tropical environments [1–4]. In China, *Ae. albopictus* is currently distributed south to Hainan Island, north to Shenyang and Dalian, west to Jingshui and Longnan, southwest to the Tibet Autonomous Region and east to most regions in China [5, 6]. Meanwhile, it is spreading northward and westward [7].

*Aedes albopictus* is an important vector of at least 26 arboviruses, including dengue virus and chikungunya virus [8]. In China, *Ae. albopictus* is the major vector of dengue fever [9], and it possesses the capacity to transmit Zika virus [10]. Since the re-emergence of dengue fever in Foshan, Guangdong Province, in 1978, dengue has been intermittently epidemic in China, and the distribution of cases has been expanding [11, 12]. In Guangzhou (23°08′N, 113°16′E), dengue cases occur every year, and *Ae. albopictus* is the vital vector [1]. In 2014, 38 036 dengue cases were reported in Guangzhou, resulting in six deaths and attracting widespread public concern [13].

Diapause is defined as “a form of dormancy that is hormonally programmed in advance of its onset and is not immediately terminated in response to favourable conditions” [14], while quiescence is “a dormancy that is elicited in direct response to unfavourable environmental conditions and is immediately terminated upon the return of favourable environmental conditions” [14]. *Ae. albopictus* undergoes photoperiodic diapause at the stage of the pharate first larva in eggs; the shortening days act as a key environmental signal of the advent of winter and cause female mosquitoes to lay diapause-programmed eggs. *Ae. albopictus* diapause, which is characterized by halted development, reduced metabolism, and increased resistance [15–17], offers a mechanism for surviving unfavourable environments, such as winter environments or poor-quality habitats. Thus, diapause behaviour contributes to the strong adaptability of the species and, thus, the potential of mosquito-borne disease transmission [18]. Hawley et al. [19] reported the first well-established *Ae. albopictus* population in Texas, USA, in the summer of 1985. They concluded that this population had arisen from trade in car tires from Asia that contained dormant *Ae. albopictus* eggs. Zhong et al. [20] conducted a genetic tracing analysis of the *Ae. albopictus* population (2001, 2011) that invaded Los Angeles, California, USA, and they speculated that photoperiodic diapause contributed to the successful colonization of the invasive *Ae. albopictus* population from Guangdong, China. Additionally, Guo et al. [21] found that the dengue-2 virus could survive in diapause eggs of *Ae. albopictus*. Furthermore, dengue-2 virus can be transmitted vertically through diapause eggs, and F1 adult mosquitoes raised from diapausing eggs can transmit dengue-2 virus to sensitive mice horizontally via biting [22].

The tropical jungle of Southeast Asia is the native habitat of *Ae. albopictus*, and the *Ae. albopictus* population from China has been found to be the oldest one [23]. In Guangzhou, Liu et al. [24, 25] observed diapause in *Ae. albopictus* on the campus of Sun Yat-Sen University upon investigating a semi-outdoor environment in the 1980s. As described by other studies during the 1980s [19, 24, 25], the common methods of diapause determination seldom excluded the possibility of quiescent eggs of *Ae. albopictus*; thus, eggs that did not hatch after being submerged in water only once and exhibited an intact embryo were considered in diapause. In addition, it is unknown whether the diapause behavior in wild populations of *Ae. albopictus* in Guangzhou has changed in the following 30 years. Moreover, based on the data collected from 2007 to 2011, the climate-driven mechanistic population model of *Ae. albopictus* with diapause, which was used to simulate *Ae. albopictus* populations, performed well in the Shanghai population, but not in the Guangzhou population [26]. Furthermore, the phenotypic plasticity of diapause in *Ae. albopictus* populations varies in the subtropical regions bordering the Tropic of Cancer. For example, populations from Hong Kong (22°15′N) in China were not sensitive to short-day photoperiods [19], whereas those from Hanoi (21°01′N) in Northern Vietnam were [27]. Consequently, we hypothesize that the *Ae. albopictus* population may present a different diapause pattern in Guangzhou (23°08′N), which is located due south to the Tropic of Cancer in the subtropic monson climate zone. Therefore, in the present study, we systematically investigated the diapause pattern of the Guangzhou population in both the laboratory and the field. The critical photoperiod (CPP), seasonal dynamics of diapause incidence, and relationships between diapause and the meteorological factors in the *Ae. albopictus* population were clarified. Meanwhile, the investigation system and statistical models for the study on *Ae. albopictus* diapause were optimized.

## Methods

### Mosquito collection and rearing

**Fig. 1:**
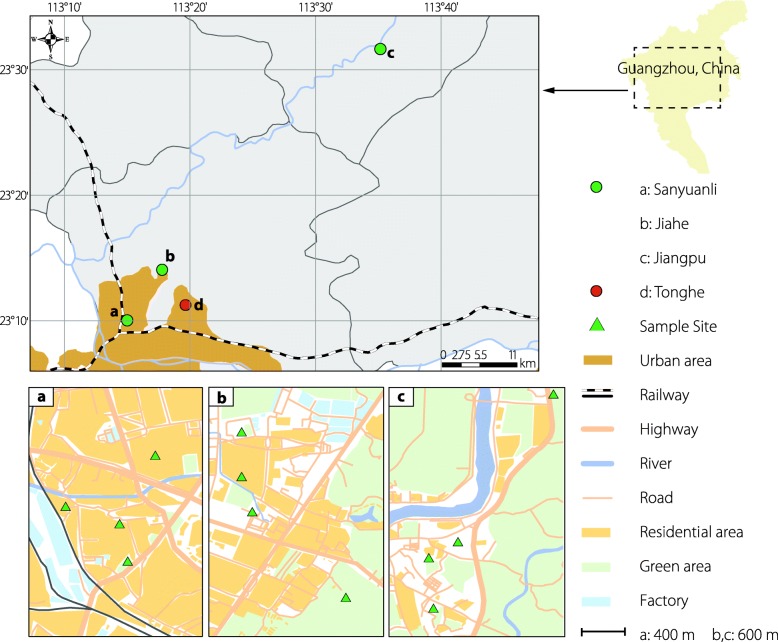
Study areas in Guangzhou, Guangdong Province, China. **a** (Sanyuanli, Yuexiu District), **b** (Jiahe, Baiyun District), and **c** (Jiangpu, Conghua District) represent the three sites at which the populations were monitored. d represents Tonghe, Baiyun District, the site at which seasonal diapause was monitored using our improved ovitraps. The green triangles indicate the four different ecological habitats (construction site, garden, residential area, and school) that correspond to the three studied sites

The Guangzhou wild population was established from individuals collected as larvae from more than ten containers located at the 12 investigation sites (shown in Fig. 1). To increase the population size, the collected individuals were reared for four generations at 28 °C and 60–80% relative humidity (RH) under a non-diapause-inducing long-day (LD) photoperiod of 16 L:8D (16 h light:8 h dark cycle). The Foshan strain [28] of *Ae. albopictus*, which was used to form the control population, was obtained from the Center for Disease Control and Prevention of Guangdong Province, China, where it has been laboratory-reared since 1981. Pumpuni et al. [29] reported that 21 °C might be the optimized temperature to induce diapause in *Ae. albopictus* populations at any given photoperiod under laboratory conditions. Thus, as optimized in previous studies, before the diapause-related experiments were performed, both the Guangzhou wild population and the Foshan strain were reared for two generations at 21 °C and approximately 80% RH under LD conditions [30, 31].

Adult mosquitoes were supplied with a 10% sucrose solution. Females were blood-fed on anesthetized mice three days after emergence [28]. Five days after blood feeding, each cage was provided with a dark oviposition cup half-filled with deionized water and containing a piece of filter paper. The paper was removed three days later and was kept wet for two days post-oviposition to allow serosal cuticle formation, which increases the desiccation resistance of the eggs [32].

### Photoperiodic diapause induction and determination and comparison of two methods for determining diapause in eggs

We set up two photoperiod smart artificial climate boxes with programmable lighting at 21 °C and approximately 80% RH. One was programmed with a LD photoperiod, i.e., a non-diapause-inducing photoperiod; the other was programmed with an diapause-inducing short-day (SD) photoperiod (8 L:16D) to induce diapause [31–33]. Three batches of larvae of each population (the Guangzhou wild population and the Foshan strain population) were reared in each artificial climate box. Mosquito rearing and oviposition were conducted as described in Mosquito collection and rearing. The eggs were maintained in Petri dishes in darkness for 10 days at 21 °C and approximately 80% RH to allow complete embryo development.

We compared two slurries used to stimulate eggs to hatch for the diapause determination: a yeast powder slurry and a food slurry (consisting of ground dog food and brine shrimp), as described by Poelchau et al. [32]. The yeast powder slurry is less expensive and easier to obtain in China than the food slurry. Diapause was induced in the two *Ae. albopictus* populations, the Foshan strain and the Guangzhou wild population, and the occurrence of diapause in the eggs was then determined using both methods at the same time. We stimulated the eggs to hatch by completely submerging individual egg paper in water in a fresh Petri dish and then re-drying them, after that the unhatched eggs were hatched again after 1 week. Then, each egg paper that had been stimulated twice was placed in a new Petri dish with Trpiš solution [32, 34] for diapause identification. After bleaching for 30 min, the eggs were observed and counted under a stereomicroscope. Embryos of unhatched eggs that presented pigmented ocelli and an egg burster and lacked abnormal pigmentation or malformation were considered to be in diapause [34]. The diapause rate was calculated according to the following formula: diapause incidence (%) = (embryonated unhatched eggs × 100) / (embryonated unhatched eggs + hatched eggs) [30, 31].

### Experimental determination of the critical photoperiod

The CPP determination was conducted under standardized laboratory conditions as in previous studies [33]. We first performed a preliminary experiment in which we tested photoperiods from 11 L: 13D to 15 L: 9D spanning Guangzhou’s minimum day length (11.5 h) and maximum day length (14.4 h) under natural conditions (Additional file 2). The value of CPP ranged between 12 and 13 h according to the preliminary experiment. Therefore, we reduced the day length span into 11–13.5 h and reduced the gradient from 1 h to 0.5 h so that we could obtain a more precise CPP value. In brief, *Ae. albopictus* collected from Guangzhou field were reared at 21 °C, 80% RH, and a LD photoperiod for 2 generations. Then, the F3 generation fourth-instar larvae and pupae were transferred to different cabinets under different photoperiod conditions: 13.5 L:10.5D, 13 L:11D, 12.5 L:11.5D, 12 L:12D, 11.5 L:12.5D, and 11 L:13D cycles, with a constant temperature of 21 ± 1 °C and 60–80% RH. At least three cages containing approximately 200 adults were established under each photoperiod. The mosquito rearing, oviposition, and diapause incidence determination were conducted as described above.

### Seasonal dynamics of the *Ae. albopictus* population in Guangzhou

#### Experimental field area

All of the field experiments involving *Ae. albopictus* were conducted in Guangzhou, the capital city of Guangdong Province, China.

The field surveys of the population were conducted in three areas that represented urban (Sanyuanli, Yuexiu District; Fig. 1, site a), suburban (Jiahe, Baiyun District; Fig. 1, site b), and rural settings (Jiangpu, Conghua District; Fig. 1, site c) in Guangzhou. Each area included four different ecological habitats (Fig. 1, green triangle in each site): construction sites, gardens, residential areas, and schools; thus, a total of 12 sites were studied. In recent years, cases of dengue fever have occurred in all three of the studied areas. Sanyuanli is an old urban area with a population density of approximately 10 000 persons/km^2^; it contains schools and old residential buildings. Jiahe is a suburban area near the urban boundary next to Baiyun Mountain; it features a mixture of residential buildings, construction sites, manufacturing facilities, and parks and has a population density of approximately 3000 persons/km^2^. Jiangpu is a rural area with a population density of < 500 persons/km^2^; its main land use types are farmland and woodland.

### Monitoring of mosquito population dynamics

The population dynamics were monitored using Mosq-ovitraps [35] and by performing aquatic habitat surveys. The Mosq-ovitrap (Southeast Industrial Co., Ltd., Guangdong, China; China patent ZL03273724.6) is composed of a cylindrical transparent plastic bottle 70 mm in diameter and 100 mm high with a well-fitting, round plastic lid that is 75 mm in diameter and 23 mm tall. The bottom of the container protrudes upward, forming an elliptical cone 20 mm high, on which filter paper, which is kept moist by water placed in the groove around the cone, is laid for the *Aedes* females to oviposit on. A network of 600 Mosq-ovitraps, 50 for each of the 12 studied sites, was set up in March 2015 and was maintained until March 2017. The distance between the individual traps was at least 25 m. The traps were placed in the same locations each month; after 4 days in the field, the traps were retrieved to our laboratory and were assessed for the presence of mosquito eggs, larvae, and adults. The population density was estimated using three indexes (MI, Mosquito-positive Index; OI, Oviposition Index; RI, Route Index). MI, OI, and RI were calculated according to the following formulae: MI = (number of ovitraps containing at least one *Ae. albopictus* adult) / (ovitraps collected from the study area) × 100; OI = (number of ovitraps containing at least one egg) / (ovitraps collected from the study area) × 100; RI = (positive breeding place) / (inspection route).

### Monitoring seasonal diapause with the improved ovitrap

Seasonal diapause was monitored at Tonghe, Baiyun District (23°23’N, 113°27′E; Fig. 1, site d) using 20 improved ovitraps; the distance between individual traps was at least 50 m. Eggs were collected weekly from 24 September 2016 to 16 November 2017 and were brought to the laboratory for counting and diapause determination. We modified the ovitraps described by others to allow more eggs to be collected from the field, especially in the winter; thus allowing diapause incidence to be calculated without bias. The improved ovitrap is an artificial container composed of a 5-L black bucket and filled with 2.5 L of tap water, which we changed weekly. In each container, a thermoplastic elastomer (TPE) strip with striations forms a ring around the inside wall and floats on the water surface (40 cm × 5 cm × 1 cm), providing a support for mosquito oviposition.

The eggs were washed carefully from each TPE strip onto filter paper using tap water. The collected eggs, which remained wet, were stored for 10 days in a humid atmosphere under complete darkness at 21 °C and approximately 80% RH [31]. Diapause was then determined using the yeast powder stimulation method as described above.

In addition, 50 Mosq-ovitraps were established near the 20 improved ovitraps from September 2016 to February 2017 at the same locations for four successive days each month. Egg number/trap/day was determined for the following six months and compared between the improved ovitraps and the Mosq-ovitraps.

### Monitoring of the hatching of overwintering eggs of *Ae. albopictus* in the field

The hatching of overwintering eggs was estimated by monitoring the first hatching larvae and pupae daily in the field at Tonghe, Baiyun District from 10 December 2016 to 30 April 2017. Ten improved ovitraps were employed, and the distance between individual ovitraps was at least 50 m. Upon pupation, the pupae in the improved ovitraps were collected using a pipette. They were then reared in the laboratory until adult emergence for species identification.

### Mosquito identification

The mosquitoes captured in Mosq-ovitraps were frozen at − 20 °C for 10 min and then identified morphologically under a stereomicroscope using taxonomic keys [36].

### Climate factors

Temperature and precipitation data were downloaded from the Guangzhou Climate Data Network (http://data.tqyb.com.cn/weather/index.jsp), and day length (i.e., daylight duration in this study) data were downloaded from the network (https://richurimo.51240.com/).

### Data analysis

The data from biological replicates are presented as the mean ± SD. Student’s *t* tests were used to compare means between two groups. A 4-parameter logistic (4PL) regression model was used to assess the association of daylight duration with diapause incidence in the laboratory [31]. We excluded two unreliable data points of diapause incidence at week_2017, 48_ (we used week_m, n_ to indicate the n^th^ week of the year m) and week_2017, 49_. A distributed lag non-linear model (DLNM) was used to capture the non-linear lagged associations between two variables [37]. Due to the limited number of data points, we used a linear function or a natural cubic spline with 1 or 2 degrees of freedom (*df*s) for the independent variables. The model with the lowest Akaike Information Criterion (AIC) value was selected. In the main analysis, a natural cubic spline with 1 *df* was used to control for the confounding effect of time. A non-linear association of day length with diapause incidence was assumed. We used lags of up to two weeks to capture the lagged effects of day length, and a linear function was used for short lags.

We explored the potential effects of temperature and precipitation on diapause incidence by assessing 16 different models (data available upon request). A likelihood ratio test was used to examine whether the associations of temperature and precipitation with diapause incidence were statistically significant.

A dose-response curve of difference in diapause incidence and day length, which indicated the cumulative effect of day length over the lag 0–2 week, was presented. We estimated the day lengths at week_2016, 43_ and week_2017, 41_, which were assumed to decline in the following two weeks at the same rate as observed in the field and consequently contributed to diapause in approximately 50% of the eggs at week_2016, 45_ and week_2017, 43_ (the weeks when the diapause incidence began to approach 50% in 2016 and 2017 in the field), respectively.

Sensitivity analyses were performed to assess the association of daylight duration with diapause incidence using a model with a natural cubic spline with 2 *df*s for time; the association of temperature or precipitation with diapause incidence was based on models indicating a statistically significant association. *P* values (two-tailed) less than 0.05 were considered to indicate statistical significance. All of the analyses were conducted using R 3.4.3 (R Foundation for Statistical Computing, VIE, AUS), SPSS 22.0 (IBM Corporation Armonk, NY, USA), GraphPad Prism 6.0 (GraphPad Software, CA, USA) and ArcGIS 10.2 (Environmental Systems Research Institute, CA, USA).

### Ethics statement

All the field surveys and collections conducted in private residential areas were performed with the consent and in the presence of the owners or residents. The study did not involve endangered or protected species.

## Results

### Photoperiodic diapause induction and determination and comparison of two methods for determination of diapause in eggs

**Fig. 2:**
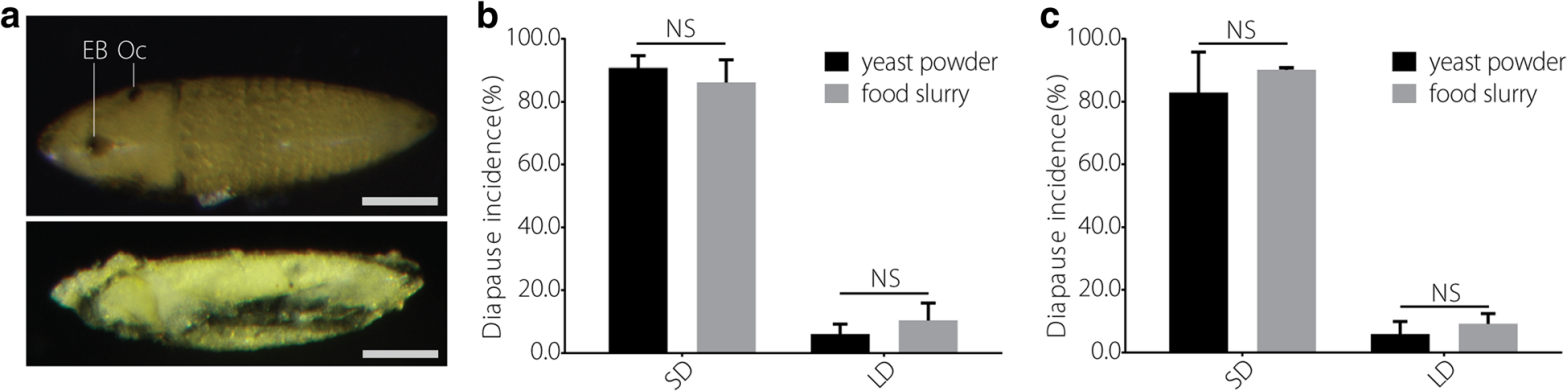
Photoperiodic diapause induction and determination and the comparison of two methods for determining egg diapause. **a** Diapause and un-embryonated eggs. **b** Diapause incidence of the Foshan strain, determined using yeast powder and food slurry. **c** Diapause incidence of the Guangzhou wild population, determined using yeast powder and food slurry. LD represents a 16 h light:8 h dark cycle; SD represents an 8 h light:16 h dark cycle. The data are from 3 biological replicates and are shown as the mean ± SD. *P <* 0.05 was considered to indicate statistical significance. NS indicates not significant. Bar = 100 μm. EB represents egg burster, and Oc represents ocelli

We found that both the Guangzhou wild population and the Foshan lab strain could be induced to enter diapause at incidences greater than 80% under the SD photoperiod in the laboratory. In addition to the food slurry, yeast powder was useful for diapause determination. Therefore, yeast powder was used for diapause determinations in the remainder of the study. In the wild population, the diapause incidence rates were 82.96 ± 12.84% and 90.23 ± 0.61% under SD conditions, as determined using the yeast powder and food slurry methods, respectively (Fig. 2c). Under LD conditions, the corresponding diapause incidence rates were 5.94 ± 3.89% and 9.07 ± 3.25%, respectively (Fig. 2c). In the Foshan strain, the diapause incidence rates were 90.82 ± 3.83% and 86.21 ± 7.10% under SD conditions, as determined using the yeast powder and food slurry methods, respectively (Fig. 2b). Under LD conditions, the corresponding incidence rates were 6.08 ± 3.06% and 10.30 ± 5.53%, respectively (Fig. 2b). The differences between two diapause determination methods were not statistically significant (*P* > 0.1) in both the Guangzhou wild population and the Foshan strain.

### Population dynamics of *Ae. albopictus* in Guangzhou

**Fig. 3:**
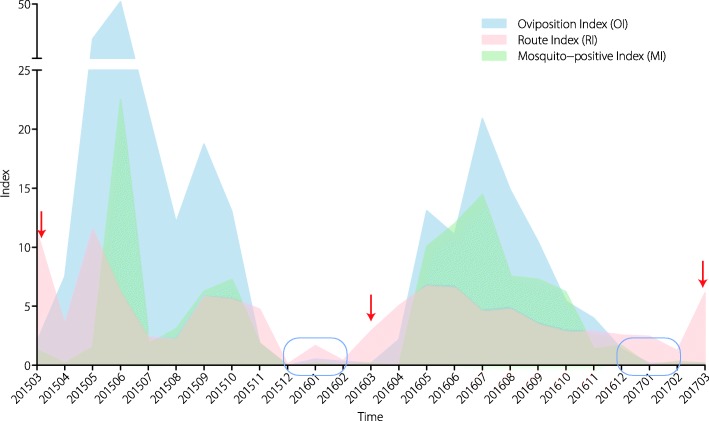
Seasonal dynamics of the *Ae. albopictus* population from 2015 to 2017 in Guangzhou, China. The average Oviposition Index, Route Index and Mosquito-positive Index values at 12 sites in Guangzhou, China, from March 2015 to March 2017 are shown. The red arrows indicate the month of March in 2015, 2016, and 2017. The blue rectangles represent the months from December to February, when all of the indexes exhibited their lowest values

In the Guangzhou subtropical habitat, the wild population of *Ae. albopictus* showed pronounced seasonal dynamics during our study period. We observed two consistent patterns in both 2016 and 2017: all of the indexes exhibited their lowest values from December to February (Fig. 3, blue rectangles), and RI relative to the larval density was the first to increase in March (Fig. 3, red arrows), before MI and OI.

### Seasonal dynamics of oviposition activity monitoring using the improved ovitraps

**Fig. 4:**
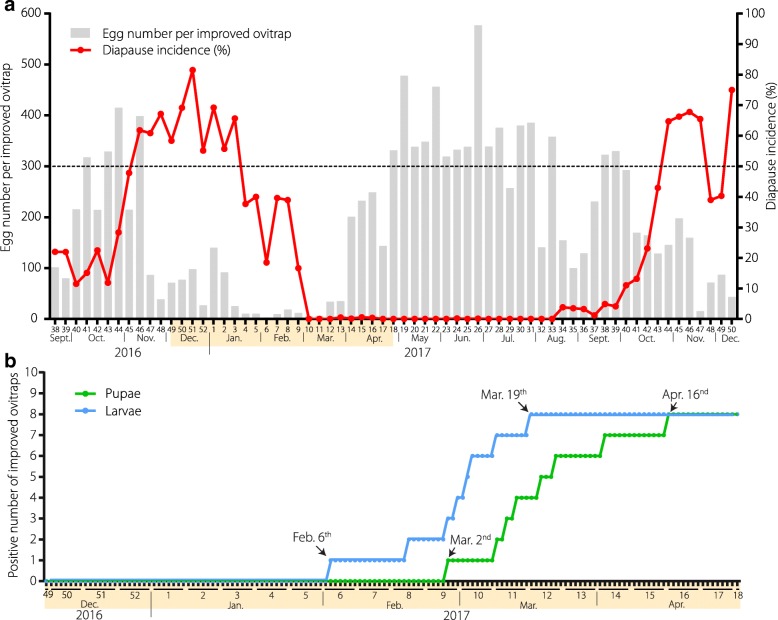
Seasonal patterns of *Ae. albopictus* diapause and hatching of diapause eggs in Guangzhou. **a** Seasonal patterns of oviposition activity and diapause incidence of *Ae. albopictus* in the field in Guangzhou, China. Twenty improved ovitraps were placed at Tonghe, Baiyun District, and eggs were collected weekly from 24 September 2016 to 16 December 2017. The numbers of eggs collected per improved ovitrap per week are shown. **b** Monitoring of the hatching of diapause eggs of *Ae. albopictus* in the field in Guangzhou, China. The hatching was monitored daily using 10 improved ovitraps at Tonghe, Baiyun District in Guangzhou from 10 December 2016 to 30 April 2017. The blue line represents the monitoring of the first larvae. The green line represents the monitoring of the first pupae. The periods with yellow backgrounds in both (**a**) and (**b**) represent the same period

Compared with the Mosq-ovitraps, the improved ovitraps present a higher efficiency for eggs collection in the field, even in winter (Additional file 3). From 24 September 2016 (week_2016, 38_), to 16 December 2017 (week_2017, 50_), a period of 65 weeks, a total of 247 525 eggs of *Ae. albopictus* were collected from the 20 improved ovitraps. A mean of 190 ± 145 eggs per ovitrap per week was obtained (Additional file 2). Egg numbers collected exhibited their lowest values during the period from late November, 2016 (week_2016, 47_) to late March, 2017 (week_2017, 13_) (Fig. 4a, gray bar graph), which was similar to the trend in OI monitored using Mosq-ovitraps (Fig. 3). The minimum quantity collected was two eggs per ovitrap during the first week of February (week_2017, 6_). The number of eggs collected greatly increased at the beginning of April and remained high thereafter, with some variations; the maximum number collected was 576 eggs per ovitrap during the last week of June (week_2017, 26_). All of the improved ovitraps were found to be egg-positive during each week of monitoring, suggesting that the egg-laying activity of *Ae. albopictus* occurs throughout the year in Guangzhou.

### Seasonal dynamics of diapause incidence in the field

A summary of the weekly diapause incidence, egg number and weather conditions is presented in Additional file 2. Diapause incidence of the *Ae. albopictus* population in Guangzhou displayed obvious seasonal dynamics. It approached 50% during the first week of November 2016 (week_2016, 45_) (Fig. 4a, red line) and peaked (81.5%) during the third week of December (week_2016, 51_), which encompasses the winter solstice, corresponding to the shortest day length of the year. The incidence then decreased, with some fluctuations; it then decreased to zero at the first week of March (week_2017, 10_) and remained there until the third week of August (week_2017, 34_). In 2017, diapause incidence approached 50% during the fourth week of October (week_2017, 43_). Meanwhile the weekly egg number decreased shortly after the diapause incidence exceeded 50% and increased again after the diapause incidence decreased to zero (Fig. 4a).

### The overwintering eggs of *Ae. albopictus* in the field start hatching in the early spring in Guangzhou

The overwintering eggs of *Ae. albopictus* were consecutively observed for 142 days. Their larvae were initially found in one improved ovitrap on 6 February 2017, and the percentages of improved ovitraps with hatched larvae reached 50% and 80% on 6 March 2017 and 19 March 2017, respectively (Fig. 4b, blue line). The pupae appeared initially in one ovitrap on 2 March 2017, and the percentage of ovitraps with pupae reached 50% and 80% on 16 March 2017 and 16 April 2017, respectively (Fig. 4b, green line).

### Association of meteorological factors with diapause incidence

**Fig. 5:**
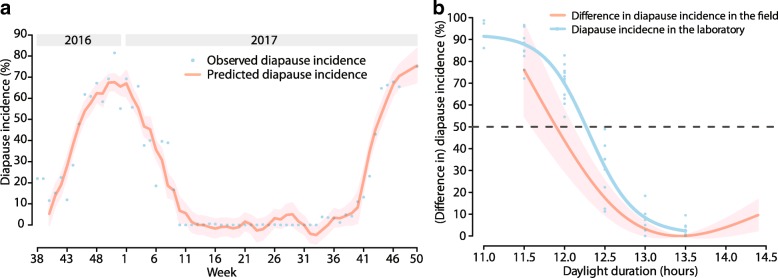
Results of DLNMs and 4PL regression model fitted to the collected data. **a** Observed and predicted diapause incidence rates in the field in Guangzhou, China during 2016–2017. **b** Dose-response curve of (the differences in) diapause incidence and daylight duration in the laboratory and in the field. The blue points in **a** represent the observed diapause incidence; the red line represents the predicted diapause incidence based on a distributed lag non-linear model with a moving average of mean temperature over a lag of 0–2 weeks. The pink area indicates the corresponding 95% confidence interval (*CI*) of the predicted diapause incidence. The blue points in **b** represent the observed diapause incidence in the laboratory; the blue line is the fitted curve based on the 4-parameter logistic regression model: $\widehat{y}=0.774+\frac{\left(92.113-0.774\right)}{1+{\left(\frac{Day\_ length}{12.312}\right)}^{44.073}}$ ($\widehat{y}$ is the predicted diapause incidence in the laboratory). The red line in **b** represents the estimated difference in diapause incidence based on the model used in **a**, with reference at 13.450 h, and the pink area is the corresponding 95% *CI* of the estimated difference in diapause incidence

After controlling for day length, the association of temperature with diapause incidence was statistically significant in some models, whereas the association of precipitation with diapause incidence was not (data not shown). The model with the smallest AIC (the model with a natural cubic spline of a moving average of mean temperature over lag 0–2 weeks but without precipitation) generally captured the temporal pattern of observed time-series of diapause incidence (Fig. 5a). The cumulative effect of day length on diapause incidence over the lag of 0–2 weeks generally decreased with day length (Fig. 5b). The fitted curve based on the 4PL regression model was consistent with these findings, indicating a negative association between daylight duration and diapause incidence in the laboratory (Fig. 5b).

The result of modelling with the smallest AIC indicated a potential non-linear association of the moving average of mean temperature with diapause incidence, with the predicted diapause incidence peaking at approximately 22.3 °C (95% confidence interval [*CI*]: 15.1–24.3) after controlling for the effect of day length (Additional file 4).

The adjusted *R*^2^ for the model with day length but without temperature was 92.8%, which was much larger than the adjusted *R*^*2*^ values for the models with temperature but without day length. Adding temperature to the models slightly improved the goodness-of-fit in some cases (Additional file 5).

The results of the model with a natural cubic spline with 2 *df* for time indicated a similar association of day length with diapause incidence in the field (data not shown). A negative association of temperature with diapause incidence was suggested by the models including minimum temperature, while the models including mean temperature and maximum temperature suggested a non-linear association of temperature with diapause incidence (data not shown).

### Daylight duration when 50% eggs were entering diapause in the laboratory and in the field

In the laboratory, 50% of the eggs entered diapause at a number of daylight hours between 12.0 and 12.5 h (Fig. 5b). According to the 4PL regression model, the CPP of this population was equal to 12.312 h (95% *CI*: 12.220–12.403) of light, which approximately corresponded to the third week of October in the field in both 2016 and 2017.

In the field, the diapause incidence began to approach 50% at week_2016, 45_ and week_2017, 43_. The day lengths at week_2016, 45_ and week_2017, 43_ were 11.909 h and 12.182 h, while the day lengths at week_2016, 43_ and week_2017, 41_ were 12.159 h and 12.466 h, respectively. Based on model 1, the DLNM with a moving average of mean temperature over a lag of 0–2 weeks, which had the smallest AIC, the predicted diapause incidence at week_2016, 45_ and week_2017, 43_ reached approximately 50%, when day length at week_2016, 43_ declined from 12.159 h to 12.111 h (95% *CI*: 12.038–12.190) and when daylight duration at week_2017, 41_ dropped from 12.466 h to 12.373 h (95% *CI*: 12.282–12.470), respectively. Therefore, it was estimated that the day lengths of 12.111 h (95% *CI*: 12.038–12.190) at week_2016, 43_ and 12.373 h (95% *CI*: 12.282–12.470) at week_2017, 41_ contributed to diapause in 50% of the eggs.

## Discussion

### Improved methods for diapause determination and seasonal diapause monitoring

In this study, we developed improved methods for diapause determination and seasonal diapause monitoring. Methods for *Ae. albopictus* diapause determination have undergone continuous improvement. Initially, the rate of diapause was measured as the rate of hatching [19, 38]; later, a method for determining diapause based on hatching stimulation was developed. Some investigators stimulate hatching by reducing the amount of oxygen in the water, usually with vitamin C [31, 39], whereas others use larval feeding solution [30, 33] to stimulate hatching. After two consecutive hatching stimulations, the embryonated eggs containing egg bursters (EB in Fig. 2a) and ocelli (Oc in Fig. 2a) are considered to be in diapause. The two consecutive hatching stimulations can largely exclude quiescent eggs. In our study, we chose to use larval feeding solution for two consecutive hatching stimulations. In addition to using food slurry consisting of dried ground dog food and brine shrimp, we used a slurry composed of the same amount of yeast powder and compared their effectiveness for diapause determination. Our results showed that both the yeast powder and the food slurry were effective for determining diapause. Compared with food slurry, yeast powder is easier to acquire, less expensive, and easier to prepare.

To survey diapause incidence in the field and to determine its dynamics, large numbers of mosquito eggs are required to obtain an accurate rate, especially in the winter, when less oviposition occurs. The improved ovitraps used in our study perform well throughout the year, which facilitated observation of the species’ habits, especially diapause, in the field. The Mosq-ovitraps [35], commonly used in China, are designed to monitor the density of mosquitoes and are characterized by their small size. They can be employed as a safe, effective and easy-to-use evaluation method for emergency mosquito control. However, the number of eggs they induce is too small to meet the requirement of monitoring diapause in the field, especially in winter (Additional file 3). Lacour et al. [31] reported the use of ovitraps made up of 3-L black plastic buckets filled with 2 L of tap water for monitoring diapause. A floating polystyrene square (5 cm × 5 cm) was added to provide a support for oviposition in their study. We further developed our ovitrap based on previous studies. Compared with others, our improved ovitrap is larger, and as a larger breeding site, it is more attractive to female *Ae. albopictus*. The oviposition support with the horizontal strips, which forms a ring around the inside wall of the ovitrap, provides the female mosquito with a larger oviposition area and reduces the loss of eggs into the water. Thus large numbers of mosquito eggs could be obtained using our improved ovitraps in both hot and humid or rainy weather.

### Characterization of *Ae. albopictus* diapause using optimized statistical models

A delay between maternal diapause induction and diapause initiation in the offspring was found in the field [31]; accordingly, there were lagged effects of day length, temperature (i.e., mean temperature, minimum temperature, and maximum temperature), and precipitation on diapause incidence in the field. A DLNM can flexibly capture the non-linear lagged associations between two variables, and DLNM models have been extensively used in ecological studies in recent years [40, 41]. In the present study, a DLNM was used to assess the associations between meteorological factors and diapause incidence in the field and to estimate the day length for 50% of eggs being programmed in diapause. We found that day length was the primary influential factor of diapause incidence and that temperature also influenced diapause incidence, whereas the influence of precipitation was not statistically significant. These results further support the idea that *Ae. albopictus* offers an ideal potential photoperiodic diapause model [14]. *Drosophila melanogaster*, the most widely used insect model in molecular biology, has a weak and highly temperature-dependent diapause response [42, 43]. In addition, we found a threshold (22.3 °C [95% *CI*: 15.1–24.3]) of the association of the moving average of mean temperature with diapause incidence. The estimated threshold might indicate the optimum temperature for inducing diapause of the *Ae. albopictus* population in Guangzhou. Interestingly, this estimated threshold was close to the commonly used diapause induction temperature of 21 °C, which was suggested by experiments. The influence of temperature on diapause induction in *Ae. albopictus* was reported in 1992; in experiments, strains tested at 21 °C and 26 °C showed clear photoperiodic responses, whereas those tested at 29 °C and above displayed greatly reduced or absent diapause incidence [29]. Since then, 21 °C has been considered an ideal temperature for inducing *Ae. albopictus* diapause in the laboratory because of the high incidence of diapause it induces [31–33, 39, 44].

CPP, the daylight duration at which 50% of individuals enter diapause, is one of the most important features of the photoperiod response [45] and marks the transition between diapause and non-diapause responses in a population [14]. The rapid evolution of CPP in the *Ae. albopictus* populations may play an important role in the mosquito’s capacities to rapidly invade new areas and to respond to global climate change [14, 33]. According to the 4PL regression model used in this study, the CPP of the Guangzhou wild population in the laboratory was estimated to be 12.312 h (95% *CI*: 12.220–12.403); in the field, it was estimated that daylight durations of 12.111 h (95% *CI*: 12.038–12.190) at week_2016, 43_ and 12.373 h (95% *CI*: 12.282–12.470) at week_2017, 41_ contributed to diapause in 50% of the eggs. Since the minimum day length in Guangzhou during our study period was 11.5 h, which is shorter than the estimated daylight duration of CPP, the Guangzhou wild population should be diapause-inducible.

There is a close relationship between CPP and latitude. Studies of *Ae. albopictus* in Japan and the United States show that for every 5° increase in latitude, CPP increases by approximately 0.5–1 h [33]. In China, the CPP of a Shanghai (31°13′N, 121°28′E) population was 13–14 h at a temperature of 25 °C under laboratory conditions [46]. The difference in CPP between the Guangzhou and Shanghai populations is consistent with this relationship.

### Different patterns of photoperiod diapause induction in subtropical regions

During our study period, day length in Guangzhou ranged from 11.5 h to 14.4 h, with an average of 12.8 h. The mean temperature, minimum temperature, maximum temperature, and precipitation were 23.1 °C, 19.0 °C, 27.5 °C, and 28.0 m (Additional file 2). In the wild population of *Ae. albopictus* in Guangzhou (23°08′N), diapause was confirmed to be induced mainly by photoperiod, consistent with previous studies [30–33]. Photoperiods have also been shown to alter developmental time, mass, and the resulting sex-specific plasticity in *Ae. albopictus* [47]*.* In most cases, diapause in *Ae. albopictus* is positively correlated with latitude. Populations collected in Beijing (39°55’N) and Shanghai (31°13’N) in China [19], Korea [19], and Japan [19, 33], all regions north of 30 °N, are sensitive to short-day photoperiods and show low hatching rates, whereas tropical Asian strains from Thailand [19], Western Malaysia (3°8’N), and Eastern Malaysia (2°48’N) [19] are not sensitive to short-day photoperiods. Notably, the capability of *Ae. albopictus* to undergo diapause varies among the subtropical regions bordering the Tropic of Cancer; populations from Hanoi (21°01’N, 105°51′E) in Northern Vietnam were photoperiod-sensitive [27], whereas strains from Hong Kong of China (22°15’N) were not [19].

In this study, by determining diapause directly rather than by hatching rate, we present evidence that *Ae. albopictus* mosquitoes of the Guangzhou wild population and the Foshan strain (23°01’N, 113°07′E) can be induced to enter diapause at a > 80% diapause incidence under SD conditions. The Foshan strain was included as a control population for two reasons. First, the strain is an established laboratory colony that has been maintained since 1984, its coordinates of origin are similar to those of the Guangzhou wild population, and its genetic background is clear. Second, 71 putative diapause-related genes have been found in the genome of the Foshan strain [28]. Additionally, diapause incidence peaked at 81% in the Guangzhou field population in 2016. Therefore, the Guangzhou wild population can be induced to > 80% diapause incidence in both the laboratory and field. These findings raise the question why Hong Kong and Guangzhou in China, and Hanoi in Vietnam, which are located at similar latitudes and experience similar climates (https://www.weather-atlas.com), have populations with different phenotypic plasticities of diapause. Diapause in Hong Kong populations in China was determined in 1987; whether the diapause capabilities of these populations have changed over the years, for example, whether a more viable diapause population has replaced a non-diapause population, is unknown. Very different responses to short day length have also been observed in populations of *Ae. albopictus* from comparable latitudes in the northern and southern hemispheres. For example, *Ae. albopictus* in Miami (25°45’N) and Card Sound, Florida (25°25’N), more temperate regions of the USA, has undergone a gradual loss of diapause, whereas *Ae. albopictus* in the two southernmost states (> 26°S) of Brazil has gradually gained diapause. This asymmetric evolution has been attributed to genetic constraints and different selective regimes [38]. The system for the study of *Ae. albopictus* diapause described by us herein may provide a practical and feasible method to gain a more complete understanding of the diapause background in these locations.

### Diapause eggs are the main form of *Ae. albopictus* for overwintering in Guangzhou

In the present monitoring in the field in Guangzhou, both adults and larvae of *Ae. albopictus* were found throughout the winter in both 2016 and 2017, but very occasionally, even in very small numbers. These data are consistent with the previous investigations in Guangzhou [48]. Meanwhile, diapause eggs composed the bulk of the *Ae. albopictus* population during the winter (from December to February of the following year) in Guangzhou in both years. Furthermore, during a period of 142 days of consecutive surveillance (from 10 December 2016 to 30 April 2017), we discovered that the overwintering eggs of *Ae. albopictus* in the field started hatching in the early spring (from early-February to mid-March) in Guangzhou, with their larvae initially found in early February and a large quantity of them found in mid-March. The diapause incidence then decreased to zero in mid-March based on our weekly observations, and the larvae density also increased in March based on our monthly surveillance. These data indicate that diapause eggs are the main form of *Ae. albopictus* for overwintering in Guangzhou, and the diapause of eggs terminates to a great extent in March, resulting in peak numbers of larvae. Therefore, for the efficient prevention and control of mosquito-borne diseases, efforts should begin in early March to eradicate the larval habitats of *Ae. albopictus* as part of the citywide public health campaigns in Guangzhou.

## Conclusions

We conducted the first comprehensive and systematic study of diapause in a subtropical population of *Ae. albopictus* in Guangzhou, China, and we established an optimized investigation system and statistical models for the study of *Ae. albopictus* diapause. Our data show that the Guangzhou population of *Ae. albopictus* can be induced to > 80% diapause incidence in both the laboratory and the field. In Guangzhou, diapause eggs are the main form for overwintering and begin to hatch in large quantities in March. Therefore, for the efficient prevention and control of mosquito-borne diseases, citywide public health campaigns in Guangzhou involving larval habitat eradication of *Ae. albopictus* should be launched in March. Meanwhile, in the present study, the CPP of this population was estimated to be 12.312 h of light according to the 4PL regression model based on the laboratory data. Based on the field observation data, daylight duration was the primary influential factor of diapause incidence, with diapause incidence generally decreasing with increased daylight duration. Day lengths of 12.111 h at week_2016, 43_ and 12.373 h at week_2017, 41_ contributed to diapause in 50% of the eggs. These findings will contribute to the prevention and control of *Ae. albopictus* and mosquito-borne diseases.

## Additional files

Additional file 1: Multilingual abstracts in the five official working languages of the United Nations. (PDF 244 kb)

Additional file 2: **Table S1.** Summary statistics for weekly egg number, diapause incidence, and weather conditions in Guangzhou, China during 2016–2017. **Figure S1.** The corresponding weather conditions of Fig. 4a during the study period. (DOCX 186 kb)

Additional file 3: **Table S2.** Egg number/day/trap as determined using Mosq-ovitraps and improved ovitraps in the field in Guangzhou between September 2016 and February 2017. (DOCX 17 kb)

Additional file 4: **Figure S2.** The predicted diapause incidence along a moving average of mean temperature over a lag of 0–2 weeks. The red line represents the predicted diapause incidence, and the shaded area is the corresponding 95% confidence interval of the predicted diapause incidence. The predicted diapause incidence was estimated for week_2017, 43_, given the same day length as that observed in the week and the previous two weeks. (DOCX 120 kb)

Additional file 5: **Table S3.** The adjusted *R*^*2*^ for models with temperature and/or day length. (DOCX 17 kb)

## Abbreviations

4PL: Four-parameter logistic
AIC: Akaike Information Criterion
CPP: Critical photoperiod
*df*s: Degrees of freedom
DLNM: Distributed lag non-linear model
LD: Long-day
MI: Mosquito-positive index
OI: Oviposition index
RH: Relative humidity
RI: Route index
SD: Short-day
TPE: Thermoplastic elastomer
