# Supplementary material for: Photoperiodic diapause in a subtropical population of Aedes albopictus in Guangzhou, China: optimized field-laboratory-based study and statistical models for comprehensive characterization
Source: Infect Dis Poverty. 2018 Aug 14;7:89. doi: 10.1186/s40249-018-0466-8 (PMC6092856; doi:10.1186/s40249-018-0466-8)
Supplement: Supplementary file 1 — Multilingual abstracts in the five official working languages of the United Nations. (PDF 244 kb) [file 40249_2018_466_MOESM1_ESM.pdf]

Translation of the abstract into the five official working languages of the United Nations

البيات المتعلق بالفترات الضوئية في تعدادٍ شبه إستوائي لحشرة الزاعجة المنقطة بالأبيض في غوانغجو: دراسة مصغرة تعتمد على العمل المخبري والميداني ونماذج إحصائية لإعطاء وصف شامل

Dan Xia, Xiang Guo, Tian Hu, Li Li, Ping-Ying Teng, Qing-Qing Yin, Lei Luo, Tian Xie, Yue-Hong Wei, Qian Yang, Shu-Kai Li, Yu-Ji Wang, Yu Xie, Yi-Ji Li, Chun-Mei Wang, Zhi-Cong Yang, Xiao-Guang Chen and Xiao-Hong Zhou

الملخص

خلفية: الزاعجة المنقطة بالأبيض هي واحدةٌ من بين أكثر 100 فصيلة جارحة في العالم وتشكل خطراً هائلاً على الصحة العامة. تشكل فترة البيات المتعلقة بالفترات الضوئية أساساً بيئياً حاسماً لتتكيف هذه الفصيلة مع بيئاتٍ غير ملائمة لها. إن الزاعجة المنقطة بالأبيض هي الناقل الوحيد الحامل لفيروس الضنك في غوانجغو، لكن نشاطات بياتها هنالك تبقى غامضة.

الوسائل: في المختبر، خضعت بودرة الخميرة ومعجون الطعام (ماء وطحين) إلى المقارنة من أجل طريقة مناسبة لتحديد البيات، كما خضعت الفترات الضوئية الحرجة (CPP) إلى الاختبار عند الإضاءة بكل من القيم 11، 11.5، 12، 12.5، 13 و13.5h. تم اختيار نموذج انحلال لوجستي بأربع بارامترات (4PL) لتقدير CPP. على الصعيد الميداني، إن التحركات الموسمية لحشرة الزاعجة المنقطة بالأبيضمن ناحية التعداد وبيات البيوض، وتفقيس البيوض في الشتاء كلها خضعت للتحقيق شهرياً وأسبوعياً ويومياً، على الترتيب. استُخدم نموذج تأخر انتشاري غير خطي (DLNM) لتقييم ارتباطات البيات بالعوامل المناخية.

النتائج: في المختبر، تعرض كل من التعداد البرّي وسلالة فوشان من الزاعجة المنقطة بالأبيض إلى التحفيز للقيام بعملية البيات بشكل معترض أكثر من 80%، ولم يُلاحظ اختلافٌ كبير (0.1 > P) بين الطريقتين لتحديد البيات. إن CPP لهذا التعداد كان مقدراً ليكون 12.312 h من الضوء. على الصعيد الميداني، كانت كل مؤشرات التعداد البرّي عند مستوياتها الأخفض من ديسمبر وحتى فبراير، وكان مؤشر Route الأول في الازدياد في شهر مارس. مثلت حادثة البيات المعروضة التحركات الموسمية. كان من المقدَّر أن أطوال اليوم التي تبلغ 12.111 h في الأسبوع43 من 2016، و12.373 h في الأسبوع41 من 2017 ساهمت في البيات لدى 50% من البيوض. تم تقدير طول اليوم على أنه العامل المناخي الرئيسي المرتبط بالبيات.

الخلاصة: إن البيات المتعلق بالفترات الضوئية لدى الزاعجة المنقطة بالأبيض في غوانغجو قد تم تأكيده وتوضيحه بشكل شامل في كل من المختبر والميدان. إن بيوض البيات هي الشكل الرئيسي للسبات الشتوي وتبدأ بالتفقيس بكميات كبيرة في شهر مارس في غوانغجو. علاوةً على ذلك، أسست هذه الدراسة أيضاً نظام تحقيق مصغَّر ونماذج إحصائية لدراسة البيات لدى الزاعجة المنقطة بالأبيض . ستساهم هذه الاكتشافات في منع سيطرة الزاعجة المنقطة بالأبيض والأمراض المنقولة عبر البعوض.

Translated from English version into Arabic by Aya AlAjjan and Sham AlRijjal, through

TRANSLATORS WITHOUT BORDERS

广州地区白纹伊蚊亚热带种群的光周期性滞育：基于优化的野外-实验室联合研究和统计学模型分析的系统性鉴定

夏丹，郭祥，胡甜，李骊，滕萍英，尹庆庆，罗雷，谢甜，魏跃红，杨倩，李树楷，谢郁，李奕基，王春梅，杨智聪，陈晓光，周晓红

引言：白纹伊蚊是全球最具入侵能力 100 种生物之一，严重威胁公众健康。光周期性滞育为其适应不利生境提供重要生物学基础。白纹伊蚊是广州地区登革病毒重要传播媒介，但该地区白纹伊蚊种群滞育行为特征尚不明晰。

方法：实验室：比较基于酵母粉和杂粮粉建立的滞育判定方法；在光照时长 11、11.5、12、12.5、13 和 13.5 h 条件下，测定临界光周期（CPP）；采用四参数逻辑回归模型推算 CPP。野外：分别以每月次和每周次监测白纹伊蚊种群密度和卵滞育率的季节消长，每日次监测越冬卵孵化；使用分布滞后非线性模型（DLNM）评估滞育率与气象因素间的相关性。
结果：实验室：广州白纹伊蚊野生株和佛山株均可被诱导滞育，滞育率>80%；两种方法所判定滞育率间无显著差异（p>0.1）；广州地区野生种群 CPP 推算为 12.312 h。 野外：种群密度相关的所有指数均在 12 月至次年 2 月呈现低谷，而次年 3 月与幼虫密度相关的路径指数先行于其他指数回升；滞育率呈现显著的季节性消长；经 DLNM 评估，周$_{2016,43}$日照时长 12.111 h 和周$_{2017,41}$日照时长 12.373 h 导致了白纹伊蚊野生种群卵滞育率达 50%，且日照时长是影响滞育的最主要气象因素。
结论：基于野外-实验室联合研究首次系统性阐明广州地区白纹伊蚊野生种群光周期性滞育行为特征。滞育卵是广州地区白纹伊蚊越冬主要方式，越冬卵在 3 月开始大量孵化。本研究建立并优化了适用于白纹伊蚊滞育生物学特性研究的调研体系和统计学分析模型。本研究为实施白纹伊蚊及蚊媒疾病有效防控提供重要依据。

Translated from English version into Chinese by Dan Xia

Diapause photopériodique parmi une population subtropicale d' *Aedes albopictus* à Guangzhou : étude en laboratoire et sur le terrain et modèles statistiques optimisés pour une caractérisation exhaustive

Dan Xia, Xiang Guo, Tian Hu, Li Li, Ping-Ying Teng, Qing-Qing Yin, Lei Luo, Tian Xie, Yue-Hong Wei, Qian Yang, Shu-Kai Li, Yu-Ji Wang, Yu Xie, Yi-Ji Li, Chun-Mei Wang, Zhi-Cong Yang, Xiao-Guang Chen et Xiao-Hong Zhou

Résumé

Contexte : *Aedes albopictus* fait partie des 100 espèces les plus invasives au monde et représente une grave menace pour la santé publique. Ce moustique connaît une diapause photopériodique qui constitue une base écologique cruciale pour son adaptation aux environnements défavorables. *Ae. albopictus* est le seul vecteur de transmission du virus de la dengue à Guangzhou, mais ses activités de diapause dans cette zone sont encore inconnues.
Méthodes : En laboratoire, de la poudre de levure et une bouillie alimentaire ont été comparées dans le but d'identifier une méthode appropriée de détermination de la diapause. La photopériode critique (PPC) a été déterminée avec des temps d'éclairement de 11, 11,5, 12, 12,5, 13, et 13,5 heures, au moyen d'un modèle de régression logistique à 4 paramètres. Sur le terrain, la dynamique saisonnière de la population d'*Ae. albopictus* a été mesurée chaque mois, la diapause des œufs chaque semaine et l'éclosion des œufs hivernaux chaque jour. Un modèle non linéaire autorégressif à retard échelonné (DLNM) a été utilisé dans le but d'évaluer les corrélations entre la diapause et les facteurs météorologiques.
Résultats : En laboratoire, la diapause de sujets de la population sauvage et de la souche Foshan d' *Ae. albopictus* a été induite avec un taux d'incidence supérieur à 80 %, sans différence significative (P > 0,1) entre les deux méthodes d'identification de la diapause. La PPC de cette population a été estimée à 12,312 heures de lumière. Sur le terrain, tous les indices de la population sauvages se sont

avérés au plus bas de décembre à février et l'indice d'itinéraire a été le premier à remonter en mars. L'incidence de la diapause présentait une dynamique saisonnière prononcée. Nous avons estimé qu'une durée de lumière diurne de 12,111 heures pendant la semaine 2016/43 et 12,373 heures la semaine 2017/41 contribuait à une diapause de 50 % des œufs. Nous avons estimé que la durée du jour était le principal facteur météorologique lié à la diapause.

Conclusions : La diapause photopériodique d' *Ae. albopictus* dans la région de Guangzhou a été confirmée et explorée de manière approfondie en laboratoire et sur le terrain. Les œufs en diapause sont le principal mécanisme d'hivernage et ils commencent à éclore en masse au mois de mars à Guangzhou. De plus, l'étude a également permis de déterminer un système d'investigation optimisé ainsi que des modèles statistiques pour l'étude de la diapause chez *Ae. albopictus*. Ces découvertes contribueront à la prévention et au contrôle de maladies véhiculées par *Ae. albopictus* et d'autres espèces de moustiques.

Translated from English version into French by Louis Gauvreau and Suzanne Assenat, through

TRANSLATORS WITHOUT BORDERS

Фотопериодическая реакция в период диапаузы (состояния покоя) у субтропической популяции комара рода *Aedes albopictus* в Гуанчжоу: оптимизированное лабораторно-полевое исследование и статистические модели для комплексной классификации

Дань Ся (Dan Xia), Сян Го (Xiang Guo), Тянь Ху (Tian Hu), Ли Ли (Li Li), Пин-Ин Тен (Ping-Ying Teng), Цин-Цин Инь (Qing-Qing Yin), Лэй Луо (Lei Luo), Тянь Се (Tian Xie), Юэ-Хун Вэй (Yue-Hong Wei), Цянь Ян (Qian Yang), Шу-Кай Ли (Shu-Kai Li), Юй-Цзи Ван (Yu-Ji Wang), Юй Се (Yu Xie), И-Цзи Ли (Yi-Ji Li), Чунь-Мэй Ван (Chun-Mei Wang), Чжи-Цун Ян (Zhi-Cong Yang), Сяо-Гуан Чэнь (Xiao-Guang Chen) и Сяо-Хун Чжоу (Xiao-Hong Zhou)

Аннотация

Справочная информация: *Комар рода Aedes albopictus* является одним из 100 наиболее инвазивных видов во всем мире и представляет собой серьёзную угрозу для здоровья населения. Фотопериодическая реакция в состоянии покоя обеспечивает ключевую экологическую основу для адаптации указанных видов к неблагоприятным условиям окружающей среды. *Комар рода Ae. albopictus* является единственным переносчиком вируса лихорадки денге в Гуанчжоу, однако его деятельность в период наступления диапаузы в данном случае остаётся невыясненной.

Методы: В лабораторных условиях для надлежащего метода определения состояния покоя были сопоставлены сухие дрожжи и питательный раствор, а также был исследован критический фотопериод при продолжительности освещения в течение 11; 11,5; 12; 12,5; 13 и 13,5 ч. Для подсчета критического фотопериода была выбрана четырехпараметрическая регресионная логистическая модель. В полевых условиях изучение сезонной динамики популяции комара рода *Ae. albopictus*, а также исследование состояния покоя на стадии яйца

и инкубирования зимующих яиц проводились, соответственно, ежемесячно, еженедельно и ежедневно. Для оценки зависимости состояния покоя от метеорологических факторов была использована нелинейная модель с распределенным лагом.

Результаты: В лабораторных условиях как дикая популяция, так и фошаньский штамм комара рода *Ae. albopictus* были введены в состояние покоя с охватом более чем 80%, при этом значительных различий ($P > 0,1$) между двумя указанными методами для определения состояния покоя не наблюдалось. По оценкам, критический фотопериод данной популяции составил 12,312 ч. света. В полевых условиях все индексы дикой популяции находились на самом низком уровне с декабря по февраль, тогда как в марте первым увеличивался индекс траектории. Распространение в состоянии покоя отобразило выраженную сезонную динамику. Было подсчитано, что продолжительность дня 12,111 ч. на 43-й неделе в 2016 году, а также продолжительность дня 12,373 ч. на 41-й неделе в 2017 году, способствовали состоянию покоя у 50% яиц. Продолжительность дня, по оценкам, считается основным метеорологическим фактором, связанным с состоянием покоя.

Выводы: Фотопериодическая реакция в состоянии покоя у комара рода *Ae. albopictus* в Гуанчжоу была подтверждена и всесторонне освещена исследованием, проведённым как в лабораторных, так и в полевых условиях. Яйца, находящиеся в состоянии покоя, являются основной формой при зимовке и в марте начинают вылупляться в значительном количестве в Гуанчжоу. Более того, данное исследование также установило оптимизированную систему исследования и статистические модели анализа состояния покоя у комара рода *Ae. albopictus*. Полученные данные внесут вклад в профилактику и контроль за комаром рода*Ae. Albopictus*, а также за заболеваниями, передаваемыми комарами.

Translated from English version into Russian by Alexander Vareiko and Liudmila Tomanek, through

TRANSLATORS
WITHOUT BORDERS

La diapausa fotoperiódica en una población subtropical de *Aedes albopictus* en Guangzhou: estudio de campo y laboratorio optimizado y modelos estadísticos para una caracterización completa

Dan Xia, Xiang Guo, Tian Hu, Li Li, Ping-Ying Teng, Qing-Qing Yin, Lei Luo, Tian Xie, Yue-Hong Wei, Qian Yang, Shu-Kai Li, Yu-Ji Wang, Yu Xie, Yi-Ji Li, Chun-Mei Wang, Zhi-Cong Yang, Xiao-Guang Chen y Xiao-Hong Zhou

Resumen

Antecedentes: el*Aedes albopictus* se encuentra entre las 100 especies más invasivas a nivel mundial y es una gran amenaza para la salud pública. La diapausa fotoperiódica proporciona una base ecológica crucial para la adaptación de estas especies a entornos adversos. El *Ae. albopictus* es el único vector que transmite el virus del dengue en Guangzhou, pero sus actividades de diapausa continúan siendo desconocidas.

Métodos: En el laboratorio, se comparó la levadura y compuestos de comida para obtener un método correcto de determinación de diapausa y el fotoperiodo crítico (CPP por sus siglas en inglés) se examinó en periodos de iluminación de 11, 11.5, 12, 12.5, 13 y 13,5 h. Se seleccionó un modelo de parámetros de regresión logística (4PL por sus siglas en inglés) para estimar el CPP. En el campo, las dinámicas estacionales de la población del *Ae. albopictus*, diapausa en huevos, y la incubación de huevos durante el invierno se investigaron de forma mensual, semanal y diaria, respectivamente.Se utilizó un modelo de no linealidad (DLNM, por sus siglas en inglés) para evaluar las asociaciones de la diapausa con los factores meteorológicos.

Resultados: En el laboratorio, tanto la población salvaje como la cepa de Foshan de *Ae. albopictus* se indujeron a diapausa con una incidencia mayor del 80%, y no se observó una diferencia significativa ($P > 0.1$) entre los dos métodos de identificación de diapausa. Se estimó que la CPP de esta población se encontraba en una luminosidad de 12.312 h. En el campo, todos los índices de la población salvaje se encontraban en los niveles más bajos desde diciembre a febrero, y el Índice de Rutas fue el primero en aumentar en marzo. La incidencia de diapausa mostró pronunciadas dinámicas estacionales. Se estimó que las longitudes durante el día de 12.111 h por semana$_{2016, 43}$ y 12.373 h por semana$_{2017, 41}$ contribuyeron a la diapausa en el 50% de los huevos. La longitud diaria se estimó como el principal factor meteorológico relacionado con la diapausa.

Conclusiones: Se confirma y esclarece con profundidad la diapausa fotoperiódica del*Ae. albopictus* en Guangzhou, tanto en el laboratorio como en el campo. Los huevos con diapausa son la principal forma en invierno y empiezan a eclosionar en grandes cantidades durante el mes de marzo en Guangzhou. Además, este estudio también estableció un sistema de investigación optimizado y modelos estadísticos para el estudio de la diapausa en el*Ae. albopictus*. Estos hallazgos contribuirán a la prevención y control de enfermedades relacionadas con el *Ae. albopictus* y las picaduras de mosquitos.

Translated from English version into Spanish by Ana Ferri and Antonella LM, through

TRANSLATORS
WITHOUT BORDERS
