# Supplementary material for: Photoperiodic diapause in a subtropical population of Aedes albopictus in Guangzhou, China: optimized field-laboratory-based study and statistical models for comprehensive characterization
Source: Infect Dis Poverty. 2018 Aug 14;7:89. doi: 10.1186/s40249-018-0466-8 (PMC6092856; doi:10.1186/s40249-018-0466-8)
Supplement: Supplementary file 2 — Table S1. Summary statistics for weekly egg number, diapause incidence, and weather conditions in Guangzhou, China during 2016–2017. Figure S1. The corresponding weather conditions of Fig. 4a during the study period. (DOCX 186 kb) [file 40249_2018_466_MOESM2_ESM.docx]

**Additional file 2:**

**Table S1.** Summary statistics for weekly egg number, diapause incidence and weather conditions in Guangzhou, China during 2016-2017

|  |  | |  | | Percentiles | |  | |
| --- | --- | --- | --- | --- | --- | --- | --- | --- |
| Variables | Mean ± SD | Minimum | | 25^th^ | 50^th^ | 75^th^ | | Maximum |
| Egg number per improved ovitrap | 190 ± 145 | 2 | | 71 | 159 | 329 | | 576 |
| Diapause incidence (%) | 23.3 ± 27.1 | 0.0 | | 0.0 | 11.5 | 45.4 | | 81.5 |
| Day length (hours) | 12.8 ± 1.0 | 11.5 | | 11.9 | 12.6 | 13.7 | | 14.4 |
| Mean temperature (°C) | 23.1 ± 5.5 | 12.9 | | 18.6 | 23.3 | 28.7 | | 31.1 |
| Minimum temperature (°C) | 19.0 ± 5.4 | 7.6 | | 14.4 | 20.3 | 24.1 | | 27.3 |
| Maximum temperature (°C) | 27.5 ± 5.6 | 15.4 | | 23.0 | 28.0 | 32.3 | | 37.1 |
| Precipitation (mm) | 28.0 ± 42.8 | 0.0 | | 0.8 | 10.2 | 36.8 | | 213.1 |


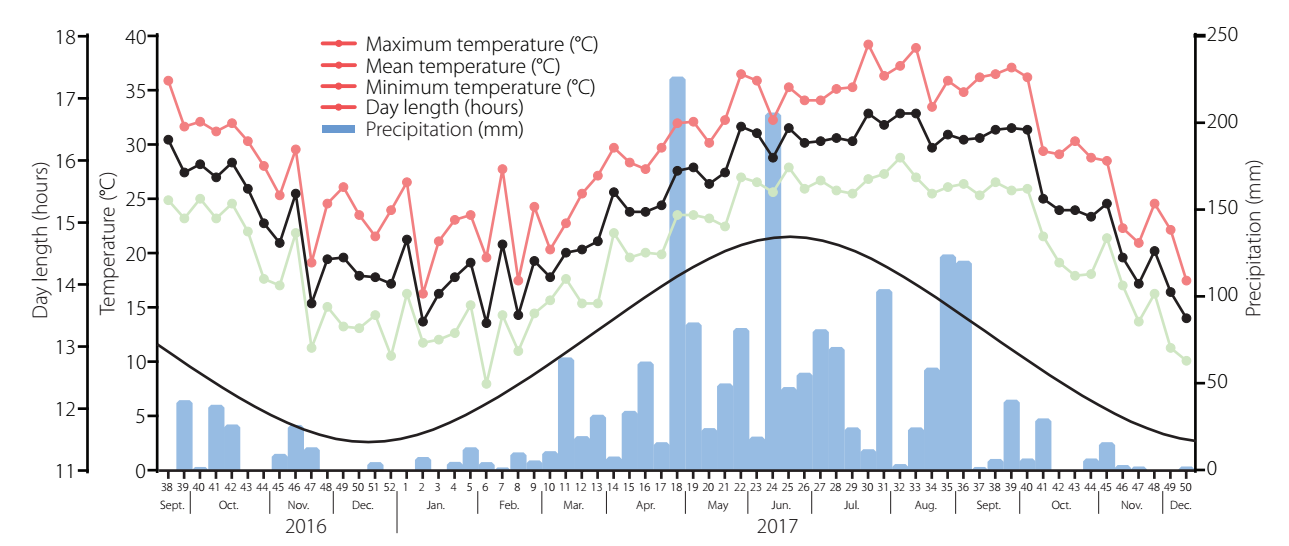


**Figure S1**. The corresponding weather conditions of Figure 4a during the study period.
