# Supplementary material for: Photoperiodic diapause in a subtropical population of Aedes albopictus in Guangzhou, China: optimized field-laboratory-based study and statistical models for comprehensive characterization
Source: Infect Dis Poverty. 2018 Aug 14;7:89. doi: 10.1186/s40249-018-0466-8 (PMC6092856; doi:10.1186/s40249-018-0466-8)
Supplement: Supplementary file 3 — Table S2. Egg number/day/trap as determined using Mosq-ovitraps and improved ovitraps in the field in Guangzhou between September 2016 and February 2017. (DOCX 17 kb) [file 40249_2018_466_MOESM3_ESM.docx]

**Additional file 3:**

**Table S2.** Egg number/day/trap as determined using Mosq-ovitraps and improved ovitraps in the field in Guangzhou between September 2016 and February 2017

|  | Trap | Sept. | Oct. | Nev. | Dec. | Jan. | Feb. |
| --- | --- | --- | --- | --- | --- | --- | --- |
| Egg number/day/trap | Improved ovitraps | 14.45 | 30.55 | 56.87 | 3.76 | 13.03 | 1.64 |
|  | Mosq-ovitraps | 0.21 | 0.00 | 0.28 | 0.07 | 0.04 | 0.00 |
