# Supplementary material for: Photoperiodic diapause in a subtropical population of Aedes albopictus in Guangzhou, China: optimized field-laboratory-based study and statistical models for comprehensive characterization
Source: Infect Dis Poverty. 2018 Aug 14;7:89. doi: 10.1186/s40249-018-0466-8 (PMC6092856; doi:10.1186/s40249-018-0466-8)
Supplement: Supplementary file 4 — Figure S2. The predicted diapause incidence along a moving average of mean temperature over a lag of 0–2 weeks. The red line represents the predicted diapause incidence, and the shaded area is the corresponding 95% confidence interval of the predicted diapause incidence. The predicted diapause incidence was estimated for week2017, 43, given the same day length as that observed in the week and the previous two weeks. (DOCX 120 kb) [file 40249_2018_466_MOESM4_ESM.docx]

**Additional file 4:**


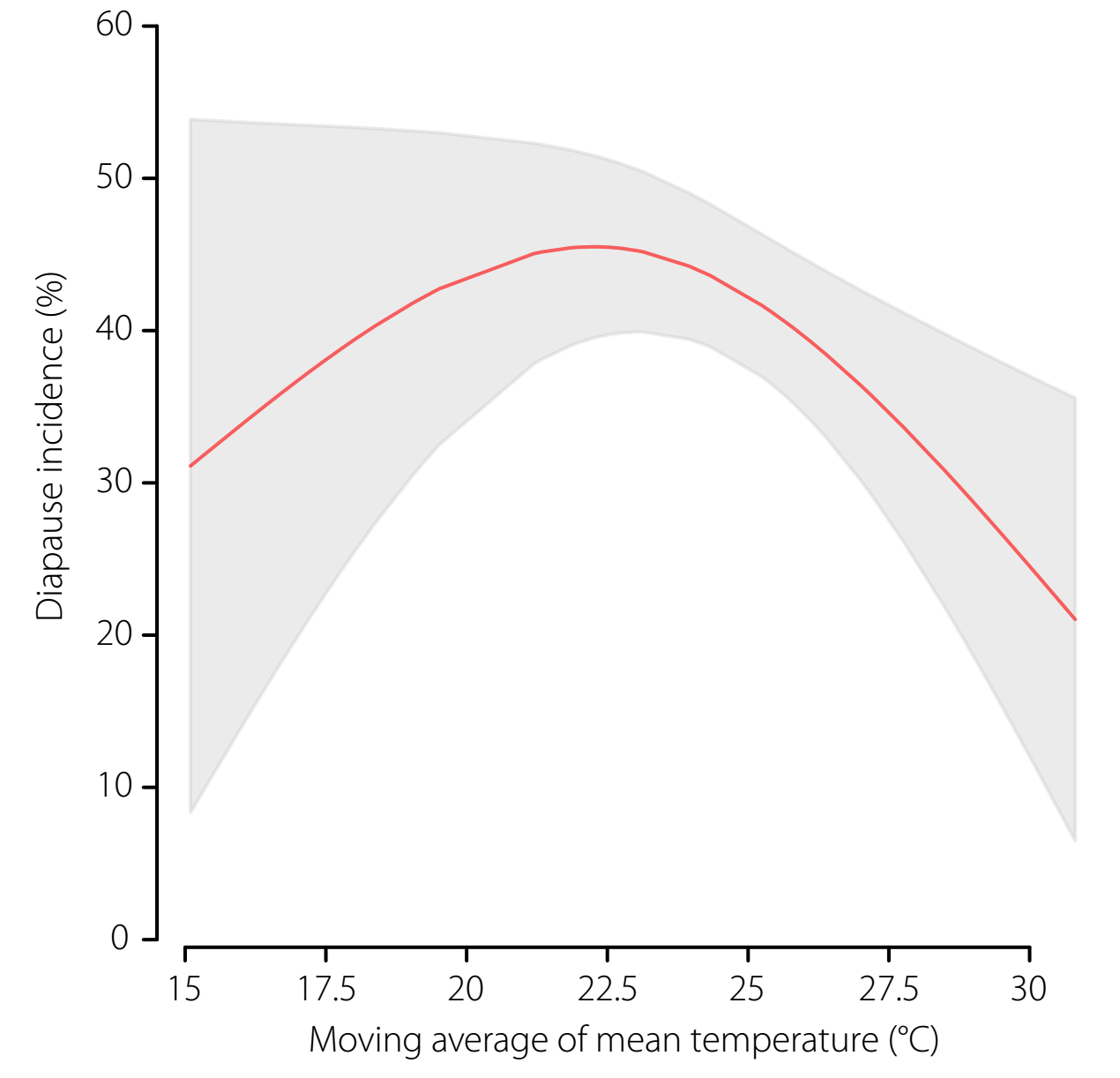


**Figure S2.** The predicted diapause incidence along a moving average of mean temperature over a lag of 0-2 weeks. The red line represents the predicted diapause incidence, and the shaded area is the corresponding 95% confidence interval of the predicted diapause incidence. The predicted diapause incidence was estimated for week_2017,43_, given the same day length as that observed in the week and the previous 2 weeks.
