# Supplementary material for: Photoperiodic diapause in a subtropical population of Aedes albopictus in Guangzhou, China: optimized field-laboratory-based study and statistical models for comprehensive characterization
Source: Infect Dis Poverty. 2018 Aug 14;7:89. doi: 10.1186/s40249-018-0466-8 (PMC6092856; doi:10.1186/s40249-018-0466-8)
Supplement: Supplementary file 5 — Table S3. The adjusted R2 for models with temperature and/or day length. (DOCX 17 kb) [file 40249_2018_466_MOESM5_ESM.docx]

**Additional file 5:**

**Table S3.** The adjusted *R^2^* for models with temperature and/or day length.

| Temperature^#^ | Adjusted *R^2^* (%) |  |
| --- | --- | --- |
|  | Without day length | With day length |
| ns(mean.t02, 2) | 40.1 | 93.7 |
| ns(min.t02, 2) | 42.1 | 93.6 |
| ns(max.t02, 2) | 36.9 | 93.6 |
| mean.t02 | 40.3 | 92.9 |
| min.t02 | 42.3 | 93.3 |
| max.t02 | 36.7 | 92.7 |
| mean.t.m1 | 49.9 | 92.9 |
| min.t.m1 | 49.4 | 93.6 |
| max.t.m1 | 45.2 | 92.5 |
| mean.t.m2 | 50.0 | 93.6 |
| min.t.m2 | 48.5 | 93.7 |
| max.t.m2 | 44.9 | 93.3 |

^#^ mean.t02, min.t02 and max.t02 were moving averages over lag 0-2 weeks for mean temperature, minimum temperature and maximum temperature, respectively. mean.t.m1, min.t.m1 and max.t.m1 represent the matrices obtained by applying a distributed lag non-linear model (DLNM) to mean temperature, minimum temperature and maximum temperature, respectively using a linear function for the variables. mean.t.m2, min.t.m2 and max.t.m2 are the matrices obtained by applying a DLNM to mean temperature, minimum temperature and maximum temperature, respectively using a natural cubic spline with 2 *df*s for the variables.
